# Dissecting the Active Site of the Collagenolytic Cathepsin L3 Protease of the Invasive Stage of *Fasciola hepatica*

**DOI:** 10.1371/journal.pntd.0002269

**Published:** 2013-07-11

**Authors:** Ileana Corvo, Anthony J. O'Donoghue, Lucía Pastro, Natalia Pi-Denis, Alegra Eroy-Reveles, Leda Roche, James H. McKerrow, John P. Dalton, Charles S. Craik, Conor R. Caffrey, José F. Tort

**Affiliations:** 1 Departamento de Genética, Facultad de Medicina, Universidad de la República, UDELAR, Montevideo, Uruguay; 2 Department of Pharmaceutical Chemistry, Pharmacology, and Biochemistry and Biophysics, University of California, San Francisco, California, United States of America; 3 Laboratorio de Interacciones Moleculares, Facultad de Ciencias, Universidad de la República, UDELAR, Montevideo, Uruguay; 4 Department of Chemistry and Biochemistry, San Francisco State University, San Francisco, California, United States of America; 5 Center for Discovery and Innovation in Parasitic Diseases, Department of Pathology, University of California, San Francisco, California, United States of America; 6 Institute of Parasitology, McGill University, Quebec, Canada; University of Queensland, Australia

## Abstract

**Background:**

A family of secreted cathepsin L proteases with differential activities is essential for host colonization and survival in the parasitic flatworm *Fasciola hepatica*. While the blood feeding adult secretes predominantly FheCL1, an enzyme with a strong preference for Leu at the S_2_ pocket of the active site, the infective stage produces FheCL3, a unique enzyme with collagenolytic activity that favours Pro at P_2_.

**Methodology/Principal Findings:**

Using a novel unbiased multiplex substrate profiling and mass spectrometry methodology (MSP-MS), we compared the preferences of FheCL1 and FheCL3 along the complete active site cleft and confirm that while the S_2_ imposes the greatest influence on substrate selectivity, preferences can be indicated on other active site subsites. Notably, we discovered that the activity of FheCL1 and FheCL3 enzymes is very different, sharing only 50% of the cleavage sites, supporting the idea of functional specialization. We generated variants of FheCL1 and FheCL3 with S_2_ and S_3_ residues by mutagenesis and evaluated their substrate specificity using positional scanning synthetic combinatorial libraries (PS-SCL). Besides the rare P_2_ Pro preference, FheCL3 showed a distinctive specificity at the S_3_ pocket, accommodating preferentially the small Gly residue. Both P_2_ Pro and P_3_ Gly preferences were strongly reduced when Trp67 of FheCL3 was replaced by Leu, rendering the enzyme incapable of digesting collagen. In contrast, the inverse Leu67Trp substitution in FheCL1 only slightly reduced its Leu preference and improved Pro acceptance in P_2_, but greatly increased accommodation of Gly at S_3_.

**Conclusions/Significance:**

These data reveal the significance of S_2_ and S_3_ interactions in substrate binding emphasizing the role for residue 67 in modulating both sites, providing a plausible explanation for the FheCL3 collagenolytic activity essential to host invasion. The unique specificity of FheCL3 could be exploited in the design of specific inhibitors selectively directed to specific infective stage parasite proteinases.

## Author Summary

The flatworm *Fasciola hepatica* is responsible for fasciolosis, one of the most common parasitic diseases of livestock worldwide, with increased incidence of human cases. When contaminated plants are ingested, infective larvae are released and transverse the gut wall before migrating to the bile ducts within the liver. Migrating liver flukes erode host tissue while adults feed on blood and they mature and release thousands of eggs. Several developmentally-regulated cathepsin L like proteolytic enzymes (FheCLs) are essential to the migrating and feeding processes. Despite being similar in structure and sequence these enzymes show specialization attacking preferentially different substrates and taking part in the diverse process of invasion, immune evasion and feeding. Our analyses reveal unique differences in activity between the major infective juvenile (FheCL3) and adult (FheCL1) enzymes, and demonstrate that the juvenile enzyme has a particular active site that allows it to degrade collagen, the main component of connective tissues. We demonstrate that a single position on the active site, residue 67, is essential to this collagenolytic activity critical for parasite invasion.

## Introduction

The common liver fluke *F. hepatica*, together with *F. gigantica*, are the causative agents of fascioliasis, a zoonosis causing huge global losses in the agricultural section by infecting more than 700 million ruminants worldwide. The disease is also recognized by the WHO as an important emerging neglected disease of humans, particularly in areas of South America, Asia, Iran and Egypt [1]. Infection with this parasite is acquired by the ingestion of plants contaminated with metacercariae, a resistant cystic form that emerges as a newly excysted juvenile (NEJ) in the duodenum, and after traversing the gut wall migrates to the liver. The parasites spend 8–12 weeks feeding on, and severely damaging, the liver parenchyma before they move into the bile ducts and become obligate blood-feeders by sucking blood through punctures in the duct walls. As in other parasites the invasion and establishment is mediated by a delicate crosstalk between molecules generated by the parasite and the host, with proteolytic enzymes being major players in this interaction [2]. Tissue migration and feeding is facilitated by the abundant secretion of proteolytic enzymes, most particularly cathepsin L cysteine proteases [3], [4].

*F. hepatica* possesses an expanded multigene family of cathepsin L-like proteases that includes at least 5 different Clan CA (papain-like) members that are developmentally regulated and play pivotal roles in parasite survival by facilitating migration, immune evasion and feeding [5], [6]. Transcriptomic and proteomic studies have demonstrated that the infective NEJ express and secrete cathepsin L3 (FheCL3) indicating that this is critical to enabling the parasite penetrate the intestinal wall [7], [8], [9], [10]. By contrast, the blood-feeding adult expresses predominantly cathepsinL1 (FheCL1), to a lesser extent, cathepsin L2 (FheCL2) and to a relatively minor extent FheCL5. FheCL1 can be involved in parasite feeding, since in vitro experiments showed it can digest hemoglobin; both FheCL1 and FheCL2 have been implicated in immune evasion based in their *in vitro* ability to cleave native immunoglobulins [11]. Correlating with the macromolecular substrates the parasite encounters at these different locations, the cathepsin L members exhibit distinct substrate specificities [4], [11].

For papain-like proteases, the evidence points to the S_2_ subsite as being most critical to defining substrate selectivity [12]. We have shown that the juvenile FheCL3 is unusual in having a particular preference for Pro residues in the P_2_ position of peptide substrates. By stark contrast, FheCL1 has a marked preference for aliphatic and aromatic residues in the P_2_ substrate position and does not readily accept Pro. FheCL2, on the other hand, exhibits an substrate preference in between these two enzymes by preferring P_2_ aliphatic and aromatic residues but also accepting Pro, although much less efficiently than FheCL3. Most interestingly, we have previously demonstrated that the preference for P_2_ Pro confers FheCL3 and FheCL2 with the rare ability to cleave native collagen [13], [14]. Only two other cysteine proteases, mammalian cathepsin K, which is involved in bone resorption by osteoclasts [15], and the ginger rhizome cysteine proteases (CP-II or zingipain, GP2 and GP3) also exhibit this high affinity for Pro in P_2_ and collagenolytic activity [16], [17].

Comparison of crystallographic structures of several Clan CA cysteine proteases allowed the identification of residues that make up the active site cleft with the selective S_2_ pocket being delimitated by residues 67, 133, 157, 158 and 205 (papain numbering) [18], [19], [20], [21], [22], [23], [24]. While variations occur in several of these positions within the *F. hepatica* cathepsin L family the residue at position 67 has been primarily implicated in P_2_ Pro accommodation by stabilizing interactions with the planar ring of Pro in the peptide substrate [20], [25]. In FheCL3 and zingipain this position is occupied by the large aromatic residue Trp while in FheCL2 and cathepsin K a Tyr is present. Structural comparisons and molecular dynamic simulations performed by us suggested that the substrate selectivity observed in FheCL3 might be due to steric restrictions imposed by the bulky aromatic residues not only at the S_2_ subsite but also within the S_3_ pocket [13], [14]. The remarkable convergence between FheCL3 and zingipain is not only restricted to Trp67 but also the close-by position 61 at the bottom of the S_3_ pocket is occupied by a large His residue. This suggested to us that together these two active site moieties could influence the capacity of the enzymes to best accommodate Pro over other aliphatic residues, and hence account for their collagenolytic activity.

To get a clear picture of the substrate specificity of the major proteases of *F. hepatica*, we used a recently developed method involving multiplex substrate profiling and mass spectrometry (MSP-MS), that provides for unbiased subsite profiling of proteases across the entire active site [26]. In addition, the P_1_–P_4_ subsite specificities were determined by Positional Scanning- Synthetic Combinatorial Libraries of fluorogenic tetrapeptides (PS-SCL), a well-established technology to study protease substrate specificity [20], [27], [28], [29]. To test the relevance of active site positions 61 and 67 in selectivity we prepared recombinant variants of FheCL3 with the specific alterations in the S_2_ and S_3_ subsites, mimicking those present in FheCL1, and the reciprocal variants of FheCL1 in an attempt to confer this protease with collagenolytic activity. All the approaches highlight the unusual and marked preference of FheCL3 for P_2_ Pro, and additionally reveal that the P_3_ pocket has a less marked but distinctive preference for Gly. The mutational analysis emphasizes the dual role of residue 67 in modulating interactions with both P_2_ and P_3_ substrate residues and its crucial importance in juvenile FheCL3 specificity and activity. Our findings provide structural insights into the molecular determinants of active site preferences of two proteases that are vital for parasite development, which might in turn prove useful in the design of strategies to control parasite infection.

## Methods

### Generation of the FheCL1 and FheCL3 active site pocket variants

Six FheCL3 and FheCL1 variants bearing substitutions at the S_2_ and S_3_ active site pockets were constructed by site-specific mutagenesis using the QuikChange Site-Directed Mutagenesis Kit (Stratagene) as indicated in Table S1. Briefly, different pairs of complementary oligonucleotides containing the base pair substitutions to be introduced in the cathepsin gene sequences were generated and used in an outside PCR reaction employing as templates clones of FheCL1 or FheCL3 in the X4-Mfα-ScPas3 expression plasmid (kindly provided by Dr. R.J.S. Baerends and Dr. J.A.K.W. Kiel, Molecular Cell Biology Lab, Groningen Biomolecular Sciences and Biotechnology Institute, University of Groningen, The Netherlands). Double variants were obtained by using plasmids bearing the single mutations as templates. The amplified modified plasmids were propagated in bacteria, sequenced to confirm the presence of the desired mutations, and then electroporated in the *Hansenula polymorpha* yeast strain for production as previously described [30].

### Production of FheCL1, FheCL3 and the enzyme variants in yeast

FheCL1 and FheCL3 recombinant proenzymes were produced in the yeast *Hansenula polymorpha* as previously described [13], [14]. Briefly, yeast transformants were cultured in 500 ml BMGY broth at 37°C to an OD_600_ of 2–6, harvested by centrifugation at 2000 g for 10 min and induced by resuspending in 50 ml of buffered minimal media (0.67% yeast nitrogen base; 0.1M phosphate buffer pH 6.0;1% methanol) for 36 hs at 30°C. Recombinant propeptidases were secreted to the culture media, and recovered by 20–30 fold concentration of culture supernatants by ultrafiltration with a 10 kDa cut-off membrane. The proenzymes were autocatalytically activated to the mature form by incubation for 2 h at 37°C in 0.1 M sodium citrate buffer (pH 5.0) with 2 mM DTT and2.5 mM EDTA, dialyzed against PBS pH 7.3 and stored at −20°C. The protein concentration was assessed by the BCA method [31]. The proportion of functionally active recombinant enzyme was determined by titration against E-64c. The enzymes variants were obtained with the same protocol used for production of FheCL1 and FheCL3.

### Multiplex substrate profiling by mass spectrometry (MSP-MS)

The enzymatic activity of FheCL1 and FheCL3 were compared by MSP-MS, a procedure designed for unbiased profiling of protease activity [26]. A highly diversified peptide library consisting of 124 synthetic tetradecapeptides containing all possible amino acid pairs and near neighbor pairs, was used to test enzymatic activity. All peptides had unmodified termini and consist of natural amino acids except Met that was substituted by norleucine and Cys omitted because of potential disulfide bond formation. The library was distributed into three pools consisting of 52, 52 and 20 peptides and diluted to 1 µM in 25 mM sodium phosphate, pH 6.0, 1 mM DTT, 1 mM EDTA. An equal volume of FheCL1 or FheCL3 in the same buffer was added to the peptide pools such that the final concentration of each enzyme was 10 nM. An enzyme-free assay was set up as a control. Assays were incubated at room temperature and aliquots were removed after 5, 15, 60, 240 and 1200 minutes. All reactions were acid quenched to pH 3.0 or less with formic acid (4% final), evaporated to dryness and reconstituted to the original volume in 0.1% formic acid. Ten µl of each time point were injected onto a 150×0.3 mm Magic C18AQ column (Michrom Bioresources) connected to a Thermo Finnigan LTQ ion trap mass spectrometer equipped with a standard electrospray ionization source. Peak lists were generated from the raw files using PAVA software (UCSF) and searched against a database consisting of all 124 peptides using Protein Prospector. Newly formed cathepsinL1 or L3 cleavage products were identified by comparison with control assays.

### P_1_–P_4_ specificity testing using a PS-SCL

The substrate specificities of FheCL1, FheCL3 and all the variants were determined using a PS-SCL [26]. Assays were performed in 0.1 M sodium phosphate buffer pH 6.0, 1 mM DTT, 1 mM EDTA, 0,01% PEG-6000 and 0.5% Me_2_SO (from the substrates) at 25°C. Aliquots of 12.5 nmol in 0.5 µl from each of the 20 sub-libraries of the P_1_, P_2_, P_3_ and P_4_ libraries were added to the wells of a 96-well Microfluor-1 flat-bottom plates. The final concentration of each compound of the 8,000 compounds per well was 15.62 nM in a 100 µl final reaction volume. The assays were performed in triplicate, the reaction was initiated by addition of the enzyme diluted in the above buffer and monitored with a SpectraMax Gemini fluorescence spectrometer (Molecular Devices) with excitation at 380 nm, emission at 460 nm and cutoff at 435 nm.

### Enzymatic assays using synthetic fluorogenic peptides

Kinetic parameters were determined in a reaction buffer containing 0.1 M sodium phosphate buffer, pH 6.0, 1 mM DTT and 1 mM EDTA at 25°C; typically final enzyme concentrations were in the 10^−9^M range, and the substrate was added after 10 min. of incubation of enzyme in reaction buffer. Enzyme concentration was determined by active-site titration with E-64c. Enzyme activity was monitored by the hydrolysis of 7-amino-4-methyl coumarin (AMC) from the synthetic peptide substrates Z-VLK-AMC and Tos-GPR-AMC. Reaction rates with different substrate concentrations (5–100 µM) were measured in duplicate as the slope of the progress curves obtained by continuous recording in a FluoStar spectrofluorimeter at 345 excitation and 440 emission wavelengths, using an AMC standard curve for product concentration calculation. Kinetic constants, *k*_cat_ and *K*_M_, were estimated by non-linear regression analysis of the Michaelis–Menten plot using the OriginPro 6.1 software.

### Digestion of type I collagen

Protein digestion was analyzed by incubating 10 µg of type I collagen from rat tail (Sigma) with 5 µM enzyme in PBS pH 7.3, 1 mM DTT and 1 mM EDTA for different times at 28°C. Digestion reactions were stopped by adding 10 µM of E64c to the reaction mixture. Fragments were separated by SDS-PAGE gels (8% acrylamide) under reducing conditions and stained with Coomassie Brilliant BlueR-250.

### Homology modeling

Homology models of FheCL3 were generated with SwissModel [32] using as principal template the crystal structure of FheCL1 (206×). Template and models were superimposed for visualization with Swiss PDBViewer version 4.1. (http://www.expasy.org/spdbv/) [33] Active site residues were identified based on the literature and confirmed by structural alignment with human cathepsin L (1MHW), human cathepsin K (1ATK) and papain (5PAD). The FheCL3 rotamers and the W67L mutant were generated with the mutate function in the PDBViewer, and selected based on rotamer score and visual inspection.

## Results/Discussion

### FheCL1 and FheCL3 multiplex substrate profiling by mass spectrometry (MSP-MS)

MSP-MS is a novel method designed to profile protease activity, based on the cleavage of a library of 124 tetradecapetides, providing theoretically unbiased information on preferences at both sides of the cleavage point [26]. The extended nature of the tetradecapeptides allow a much more natural interaction across the protease active site providing a detailed picture of the contribution of the S and S' subsites that accommodate the substrate. The characteristics of the S' sites are generally poorly known mainly because most substrates used for enzymatic profiling place a fluorophore or chromophore in the P_1_′ position, a moiety very unlike any amino acid that the enzyme can normally accept in that pocket.

**Figure 1:**
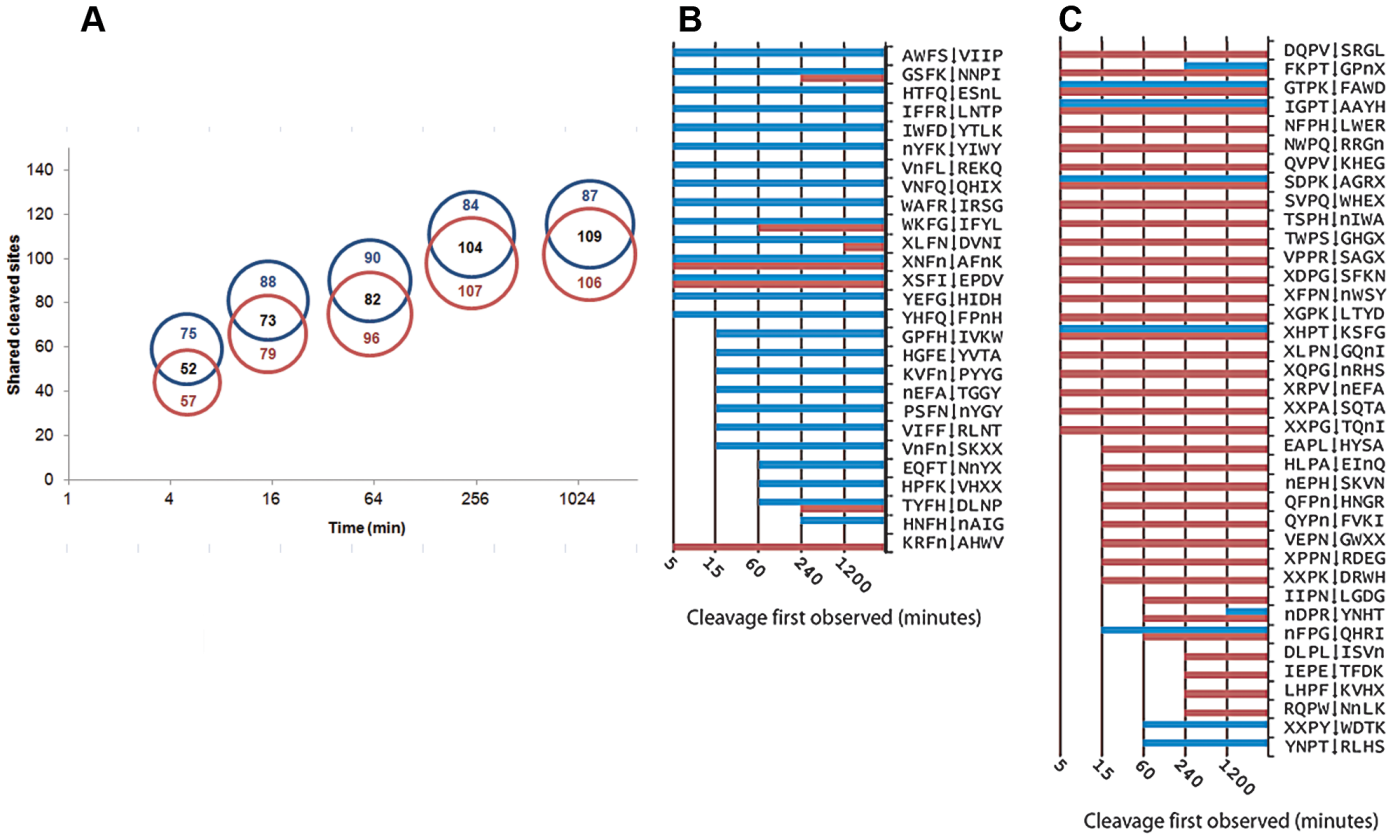
Differential cleavage of FheCL1 and FheCL3 using a multiplex combinatorial library (MSP-MS). (A). Proportional Venn diagrams showing the number of shared and exclusive cleavage sites for FheCL1 (blue) and FheCL3 (red) obtained by MSP-MS at different incubation times (5, 15, 60, 120 and 2400 min, (time axis scale is log_2_). (B) A list of all substrates containing Phe in the S_2_ position cleaved by either FheCL1 (blue) and/or FheCL3 (red) The time that cleavage was first observed is illustrated by the corresponding bar charts (C) List of all substrates cleavage points containing Pro in the S_2_ position cleaved by either FheCL1 (blue) and/or FheCL3 (red) at different times as indicated.

FheCL1 or FheCL3 were added separately to the library and all the cleaved peptides were identified at time intervals by mass spectrometry. While both enzymes cleaved at more than 170 sites after one hour incubation, FheCL1 had produced approximately 75% within five minutes of the reaction and >95% by 15 minutes. Notably, compared to FhCL1, FheCL3 produced relatively fewer cleavages at early time-points, while minor cleavages still occur for up to 20 hours of reaction, indicating differences in the ability to accommodate substrates (Figure 1 A and Figure S1). Significantly, only approximately half of the cleavage sites identified at any time were cleaved by both enzymes, leaving many that were exclusive for either FhCL1 or FhCL3 (Figure 1 B, C). A good example of this differential cleavage is offered by Peptide#38 where FheCL1 cleaves once between T∧F (EAWMT∧FIVPPRSAG) but FheCL3 cleaves twice between W∧M and R∧S (EAW∧MTFIVPPR∧SAG) and never cleaves between T∧F even after 1200 minutes incubation (data not shown).

**Figure 2:**
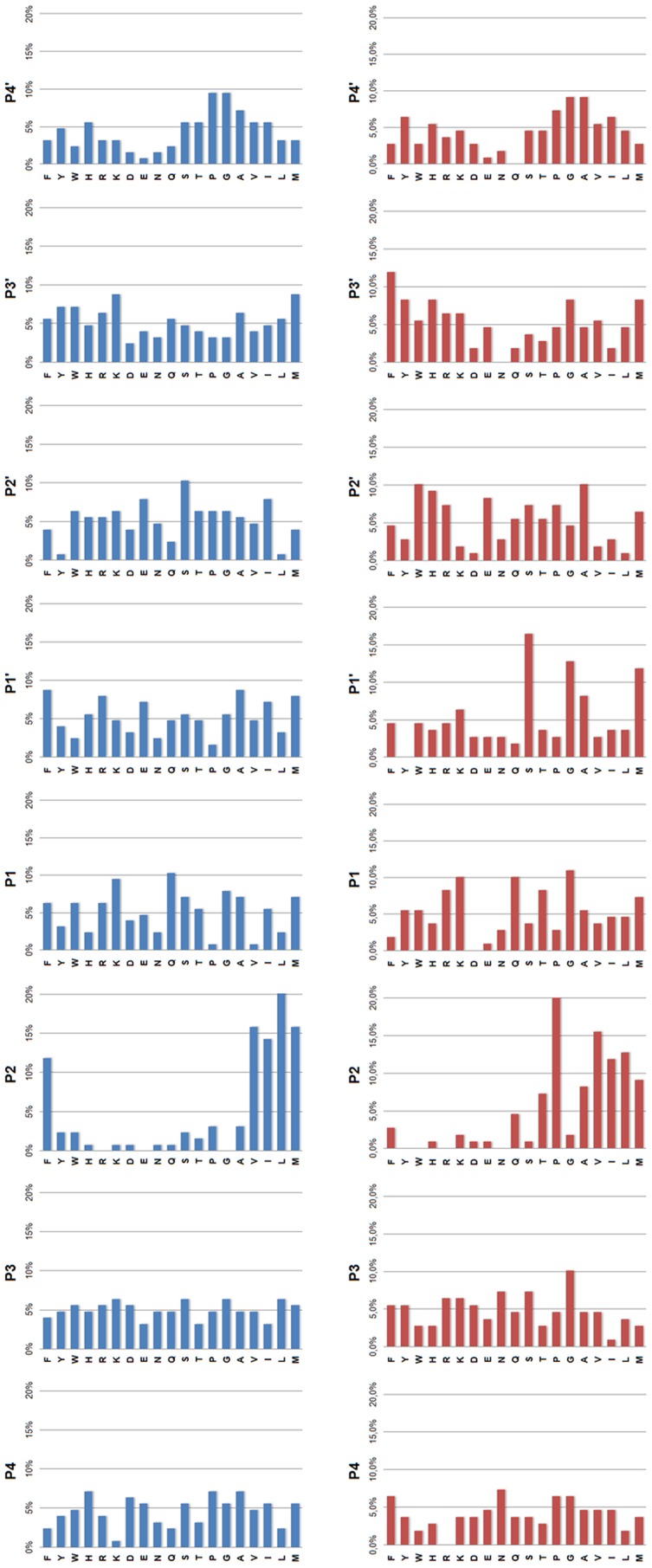
Profiling of the P_4_–P_4_′ substrate specificity of FheCL1 and FheCL3 using a multiplex combinatorial library (MSP-MS). Frequency of amino acids found at positions P_4_–P_4_′ of cleavage sites after 5 min incubation with FheCL1 (top) or FheCL3 (bottom). Results are expressed as percentage per site. The amino acid frequency at each position within the tetradecapetide library ranges from 4.2% to 6.8%. Met is substituted by norleucine in the library.

The positional analysis indicate that the substrate specificity in both FheCL1 and FheCL3 is dominated by the amino acid at P_2_ consistent to what is known about clan CA cysteine proteases [12] (Figure 2). The substrate signature at this position showed that besides aliphatic residues that can be accommodated by both enzymes, FheCL1 can readily accept Phe at P_2_ but has very low tolerance for Pro, while FheCL3 is the opposite (Figure 1B–C). In fact the preferred amino acids at this position are Leu and Pro for FheCL1 and FheCL3, respectively, confirming our previous studies using short fluorogenic peptides [13], [14]. The profile also shows that both enzymes share a strong selection against charged P_2_ residues (Figure 2). Also on the non-prime side, the juvenile enzyme, has a slight preference for Gly in the S_3_ pocket (Figure 2). This S_3_ preference is more noticeable at early digestion times (5 min. reaction), while other residues can be progressively accommodated at this site as the length of the incubation increases (Figure S1).

On the prime side of FheCL3, substrate preference is dominated by the P_1_′ site and shows a preference for Ser, Gly and to a lesser extent Met (norleucine) and Ala (Figure 2). Previous reports using internally quenched penta or heptapeptide substrates investigated the prime side preferences for papain and mammalian cathepsins B, L, S and K and showed that a broad range of amino acids were accommodated in these subsites [34], [35], [36]. However, while subtle differences were noted between the enzymes none of them can be considered as major contributions to specificity, except for a slight preference of hydrophobic moieties in papain P_3_′ [34], and a general avoidance of Pro at P_1_′ [36]. Our data confirmed the avoidance of Pro, and highlights the preference of FheCL3 for Gly and Ser, a feature that might be relevant for the enzyme's ability to degrade collagen helical domains.

### FheCL1 and FheCL3 active site preferences based on PS-SCL

Whereas the MSP-MS assay offers a more “natural” way of determining substrate specificity because the longer linear peptides are more like the loop regions found in protein substrates, Positional Scanning- Synthetic Combinatorial Libraries (PS-SCL) offer increased diversity for the study of P_4_ to P_1_ interactions since they comprise a collection of all possible fluorogenic tetrapeptides. This methodology has been widely used in the characterization of cysteine proteases [27], [28], and profiles of adult liver fluke proteases are known [20], [29]. The PS-SCL profile for the recombinant FheCL1 used in this work is practically identical with that reported by Stack et al. [20], independently supporting the accuracy of this tool in assessing enzyme specificity (Fig. S1). Furthermore, despite the differences in the methodological approaches, the PS-SCL results are generally consistent with the MSP-MS observations.

**Figure 3:**
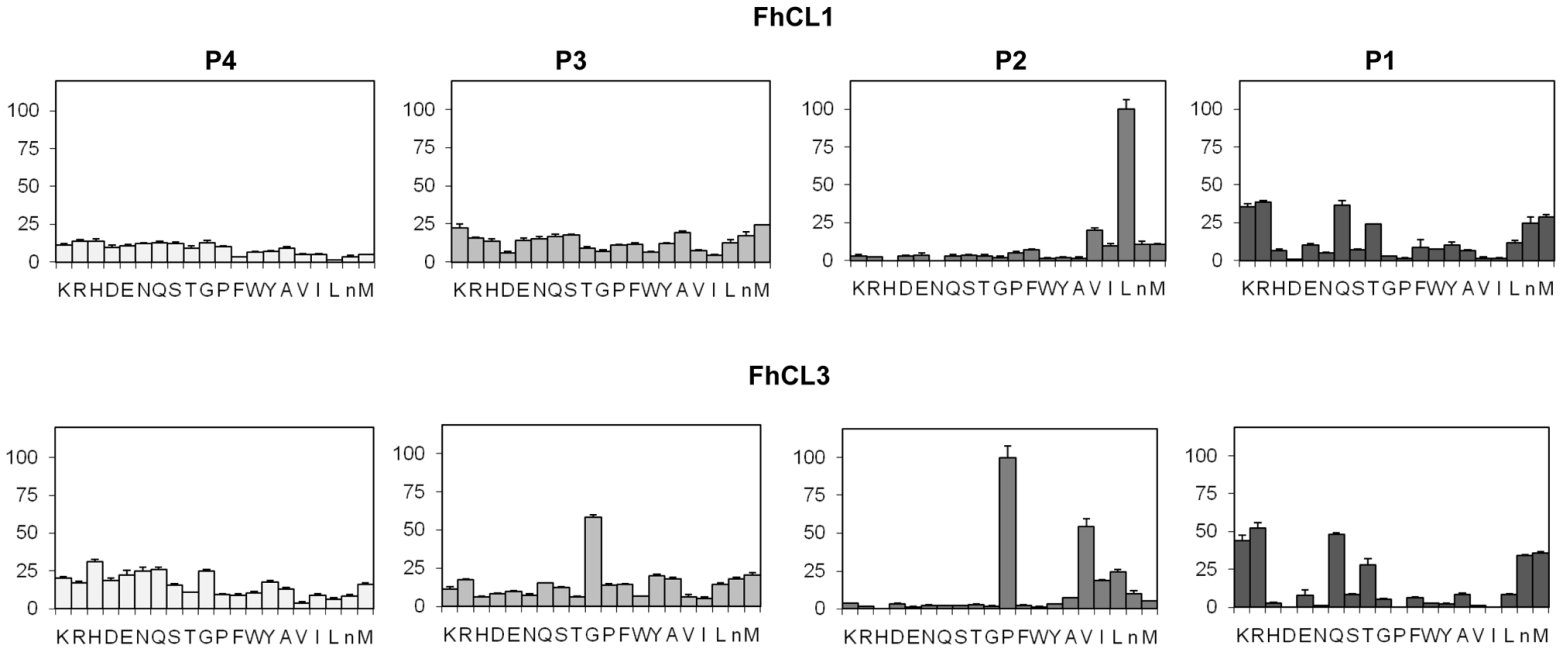
Profiling of the P_1_–P_4_ substrate specificity of liver fluke cathepsin Ls using PS-SCL libraries. The activity against the substrates is represented relative to the highest activity of the library with each enzyme (namely Leu and Pro at P2 for FheCL1 and FheCL3, respectively, n = norleucine). The error bars display the standard deviation from triplicate experiments.

FheCL1 displays a typical papain-like cysteine protease profile with S_2_ predominance, *i.e.* marked preferences for aliphatic residues, particularly Leu, at this position. Some minor selectivity can be found for the S_1_ interactions, where the basic residues Arg and Lys, together with Gln, Thr and Met are preferred. In contrast, the S_3_ and S_4_ pockets show a broad specificity completing a picture similar to that found by the MSP-MS analysis (Figure 3).

The most obvious difference between FhCL1 and FheCL3 are the very distinct profiles observed for the P_2_ and P_3_ residues. The FheCL3 S_2_ pocket can accommodate Pro very readily, accepting it twice better than Val and four times better than Leu. In addition, unlike most known cysteine proteinases, the S_3_ pocket of FheCL3 demonstrates selectivity, specifically for Gly (Figure 3). Consistent with the PS-SCL data the MSP-MS results at 5 min of digestion shows FheCL3 has a preference for Gly in P_3_ (Figure 2), and as the reaction proceed other amino acids are also accommodated in S_3_ as indicated by an increased frequency in later times. This effect is expected since in the MSP-MS assay all peptides are mixed and assayed together, consequently the preferential cleavages would be observed early in the reaction. Selectivity at S_3_ is relatively rare, although PS-SCL studies have shown that the plant enzymes papain and bromelain have a noticeable preference for Pro at P_3_ [27].

Our previous studies showed that FheCL2 also has a slightly increased preference for Gly at P3, and an augmented acceptance of Pro at P2 although maintaining Leu as the preferred residue in this position [20]. Therefore, FheCL2 active site appears to have intermediate characteristics between FheCL1 and FheCL3, both at S2 and S3 subsites (compare Figure 3 with that of Stack et al. [20] http://www.jbc.org/content/283/15/9896.full.pdfhtml). The PS-SCL profile of FheCL5, an enzyme secreted in very low abundance by adult *F. hepatica*, has also been reported and is more similar to FheCL1 with strong Leu preference at P2, although it has the unique ability to accept Asp [29]. These results support the idea of functional divergence and specialization of the different members of the liver fluke cathepsin L family occurred following several gene duplications as proposed by phylogenetic analysis [5], [6].

### FheCL3 variants in S_2_ and S_3_ active site pockets

**Figure 4:**
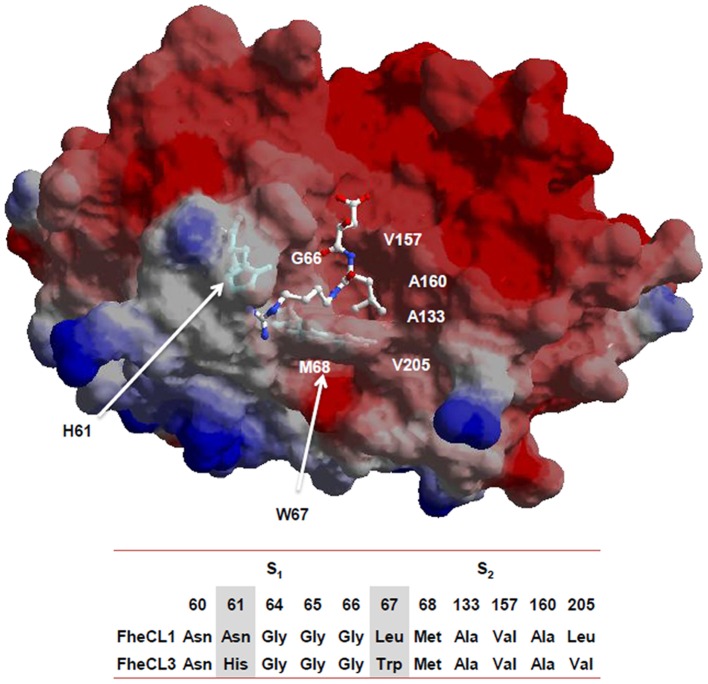
Residues contributing to substrate binding. Model of mature FheCL3 showing the S_2_ and S_3_ subsites of the active site: the His61 and Trp67 residues that were mutated in the present study are highlighted as sticks. The E64 inhibitor complexed with human cathepsin K (1ATK) was superimposed to facilitate viewing the active site cleft. The image was generated wth SPDBviewer [33] The residues constituting the active site S_2_ and S_3_ subsites of FheCL1 and FheCL3 are indicated below the molecular representation.

**Figure 5:**
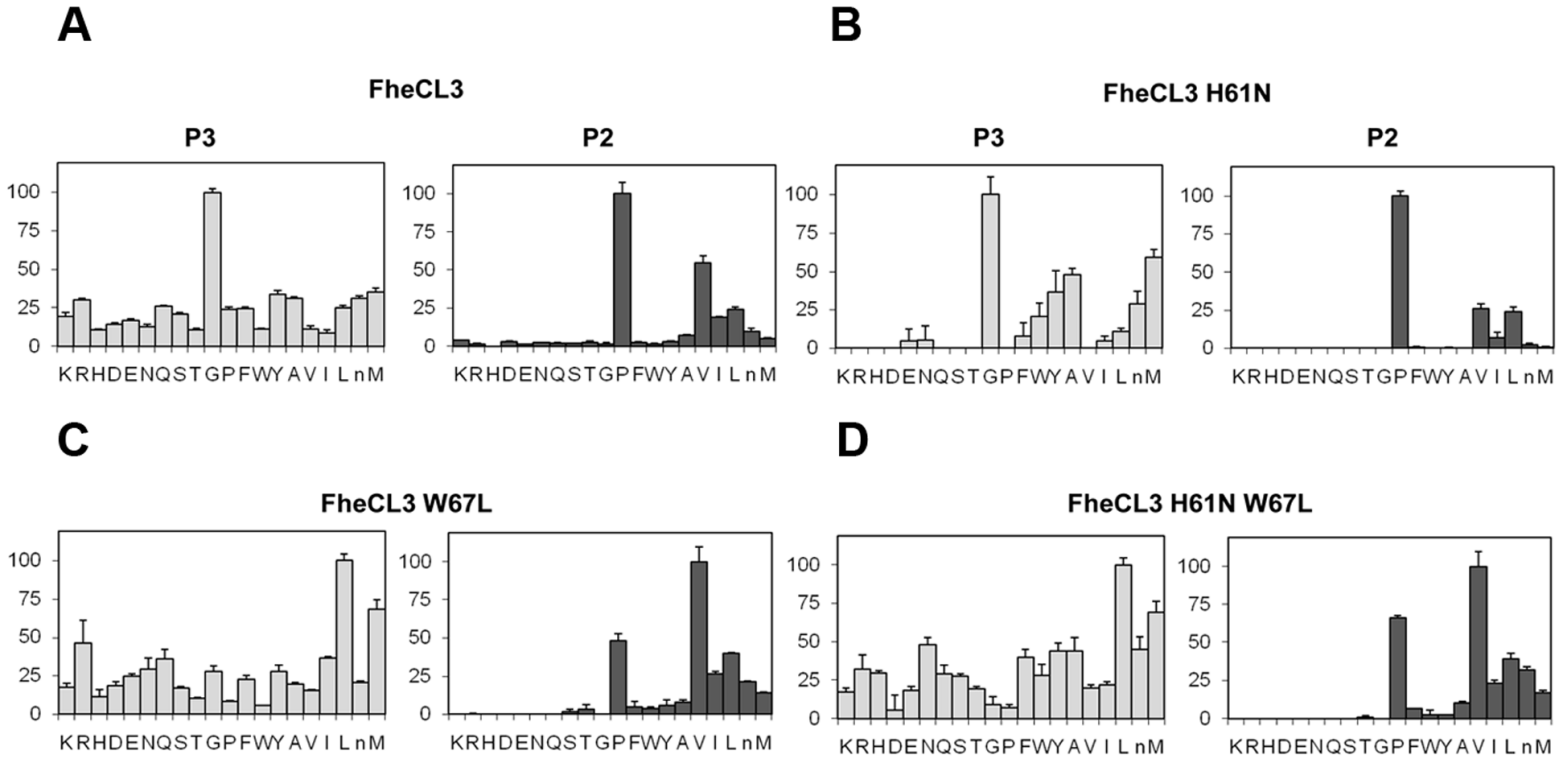
PS-SCL Profiling of the P_2_ and P_3_ substrate specificity of FheCL3 to FheCL1 enzyme variants. The activity against the substrates is represented in the *y* axis relative to the highest activity of the library, whereas the x axis shows the different amino acids using the one-letter code (n = norleucine). Error bars display the standard deviation from triplicate experiments. (A) FheCL3, (B) FheCL3 H61N (C) FheCL3 W67L (D) FheCL3 H61N W67L.

Since non-prime side differences in specificity between *Fasciola* cathepsin Ls are mainly restricted to S_2_ and S_3_, we investigated the contribution of the variable residues lining those sites by mutation analysis. These pockets differ only at three positions: 61, 67 and 205 located at the bottom of the S_3_ pocket, at the hinge of subsites S_2_ and S_3,_ and at the bottom of the S_2_ subsite, respectively (Figure 4). The first two variations involve amino acids with different properties, while the third involves a substitution between similar aliphatic moieties. Based on these observations, we changed residues 61 and 67 of FheCL3, for those present in FheCL1, generating the variants FheCL3 H61N, FheCL3 W67L and a double mutant bearing both substitutions. Their preferences at P_2_ and P_3_ were assessed with the PS-SCL approach. FheCL3 H61N showed only a subtle change in enzyme specificity, decreasing Gly preference in relation to the other amino acids as has been predicted (Figure 5 B). The FheCL3 W67L variant resulted in a marked reduction in the preference for Pro at P_2_ compared to FheCL3, while simultaneously increasing the aliphatic residues preferences and making Val the most favorably accommodated residue. Importantly, we found that the FheCL3 W67L variant also altered P_3_ specificities, changing the Gly preference to an increased preference for Leu (Figure 5 C).The double mutant enzyme, FheCL3 H61N/W67L, presented S_2_ and S_3_ profiles similar to the single FheCL3 W67L change (Figure 5 D).

### Effect of FheCL1 active site pocket residue mutations

**Figure 6:**
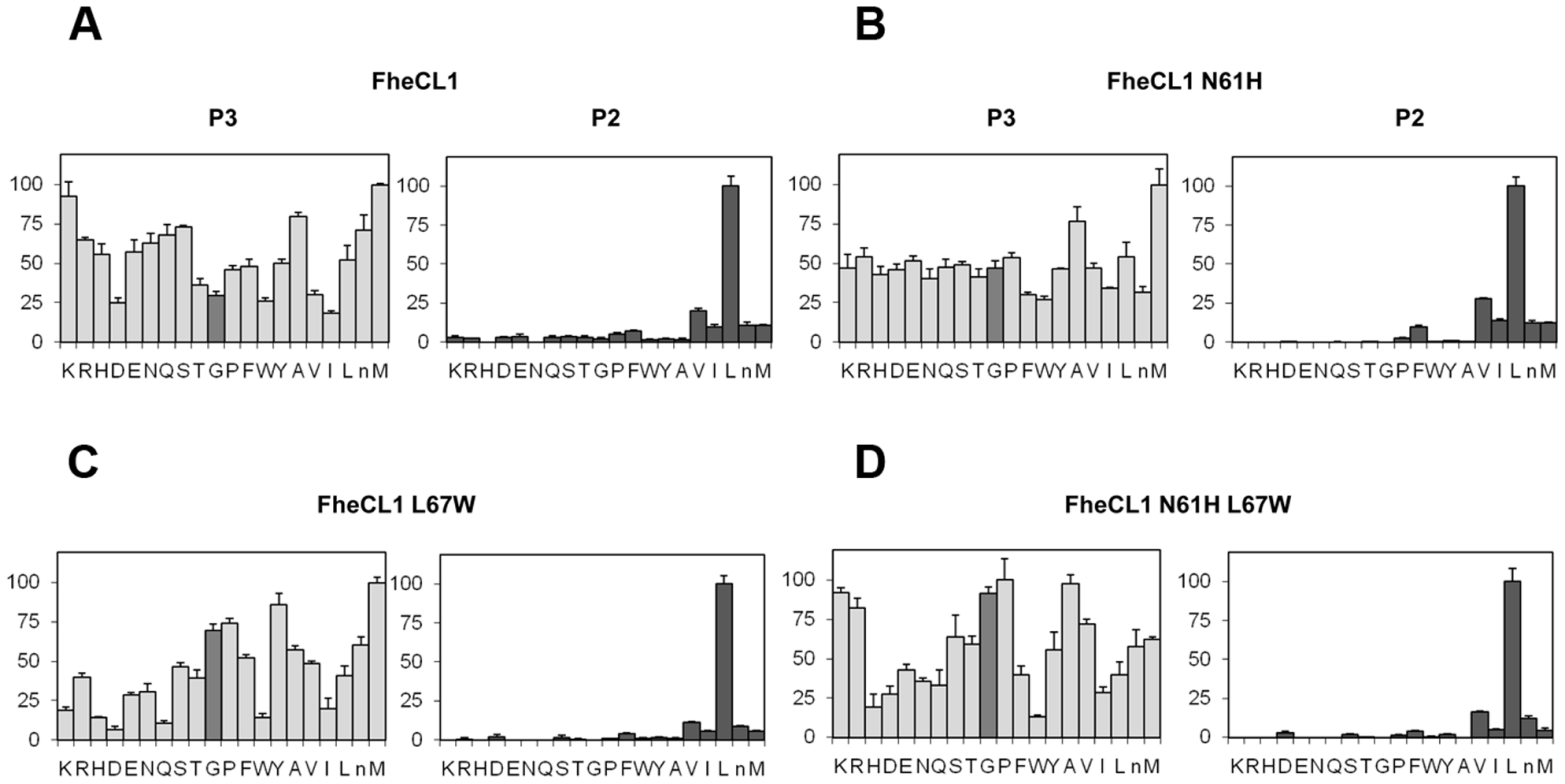
Profiling of the P_2_ and P_3_ substrate specificity of FheCL1 to FheCL3 enzyme variants using PS-SCL libraries. The activity against the substrates is represented relative to the highest activity in each sub-library (hydrolysis rate for Leu and Met fixed peptide pools at P_2_ and P_3_, respectively, are taken as 100%), whereas the x axis shows the different amino acids using the one-letter code (n = norleucine). The bar corresponding to Gly is highlighted to assist visualization. Error bars display the standard deviation from triplicate experiments. (A) FheCL1, (B) FheCL1 N61H (C) FheCL1 L67W (D) FheCL1 N61H L67W.

To complete the picture, we engineered the FheCL1 S_2_ pocket to resemble that of FheCL3 by replacing the key residues at positions 61 and 67 (Figure 4). Based on the PS-SCL neither of the changes introduced could modify FheCL1's preference for Leu at P_2_, nor increase significantly its acceptance of Pro in that position (Figure 6). However, the substitution of Leu67 to Trp did slightly increased FheCL1's acceptance of Gly in P_3_, either in the single change variant (Figure 6 C) or in the double mutant (Figure 6 D). Furthermore, in these Trp-containing variants (FheCL1 L67W, and FheCL1 N61H/L67W), the preference for Pro at P_3_ increase in comparison with the wild type enzyme (Figure 6 C–D), suggesting that the change is restricting S_3_ to small residues. The N61H single change imparts minor effects on S_3_ selectivity, suggesting that the entrance and not the bottom of the S3 pocket is crucial for selectivity (Figure 6 B).

Taken together our data shows that a single change at position 67 is sufficient to strongly reduce the unique specificities of FheCL3 at both S_2_ and S_3_ sites, and moreover, rearrange the whole active site pockets contribution to substrate recognition. Modifications at this position in FheCL1 had little effect on substrate specificity. Therefore FheCL1's preference for Leu at P_2_ seems to be robust and does not depend only on the residues lining the S_2_ or S_3_ pocket that were evaluated in this work. Different effects of modifications at position 67 have already been reported, in mammalian cathepsins [25], [37] and in the liver fluke proteases [29], [38] but these studies in general did not analyzed the possible contributions of the residues occupying the S_3_ pockets.

### Kinetic analysis of the cathepsin mutants

To support the data we observed with PS-SCL, we investigated the enzyme kinetics of the parent enzymes and their variants using two fluorogenic tripeptide substrates, Z-VLK-AMC and Tos-GPR-AMC, which are representative of the FheCL1 and FheCL3 subsite preferences. The calculated kinetic parameters *K_M_*, *k_cat_* and *k_cat_/K_M_* and the variation imposed by the diverse variants examined are presented in Table 1. We found that substitutions made at the active site residue 67 of FheCL3 resulted in a marked reduction in enzyme efficiency for both substrates (this was also seen with the PS-SCL). Compared to the parent enzyme, FheCL3 W67L exhibited a drastic diminution in specificity towards Tos-GPR-AMC (1440-fold), predominantly due to a major reduction in the catalytic turnover constant *k_cat_*. A less pronounced, though also large (35-fold) decrease in specificity towards Z-VLK-AMC (Table 1) was observed. The double variant FheCL3 H61N/W67L presented a profile very similar to the FheCL3 W67L single mutant, suggesting a minimal contribution from the H61 in the S_3_ subsite.

**Table 1:** Kinetic parameters of FheCL1, FheCL3 and the modified enzymes over two different substrates.

*Enzyme*	*Z-VLK-AMC*			*Tos-GPR-AMC*			
	*k_cat_ (s^−1^)*	*K_M_ (uM)*	*k_cat_/K_M_ (M s^−1^)*	*k_cat_ (s^−1^)*	*K_M_ (uM)*	*k_cat_/K_M_ (M s^−1^)*	*VLK/GPR k_cat_/K_M_ ratio*
FheCL1	2.32±0.120	3.94±0.93	588889	0.0350±0.0060	15.10±6.70	2355	250∶1
FheCL1 N61H	5.44±0.280	8.94±1.39	608391	0.0380±0.0040	11.76±4.30	3227	188∶1
FheCL1 L67W	0.78±0.060	10.58±2.40	73378	0.0340±0.0050	10.90±4.60	3148	23∶1
FheCL1 N61H L67W	1.30±0.130	12.01±2.90	108243	0.0310±0.0001	3.20±0.08	9615	11∶1
FheCL3 W67L	0.03±0.003	44.80±10.60	590	0.0053±0.0005	5.20±1.80	1017	1∶2
FheCL3 H61N W67L	0.03±0.004	28.70±8.60	1037	0.0072±0.0005	6.70±1.90	1072	1∶1
FheCL3*	0.33±0.010	15.70±1.80	20760	6.0000±0.4800	4.10±0.90	1464300	1∶70

* FheCL3 kinetic parameters were taken from Corvo *et al*, 2009 [14].

When analyzing the variations in the S_2_ pocket of FheCL1 we found that the variant FheCL1 L67W showed a decrease in specificity for peptides with Leu in P_2_ (Z-LR-AMC or Z-VLK-AMC). These were 8 times lower predominantly due to a decrease in the *k*_cat_ of the modified enzyme. This substitution only slightly increased the activity of FheCL1 towards Tos-GPR-AMC and hence the FheCL1 L67W variant did not nearly approach the specificity observed by FheCL3 for this substrate (Table 1). The FheCL1 S_3_ pocket replacement, FheCL1 N61H (like in FheCL3) did not alter the specificity of the enzyme towards Z-VLK-AMC and resulted in a slight increase (1.4-fold) in its activity towards Tos-GPR-AMC, likely due to a better accommodation of Gly at P_3_ which would be consistent with the observations of the PS-SCL analysis (Figure 6).

Consequently, despite finding the expected variations in Z-VLK-AMC and Tos-GPR-AMC activity in FhCL1mutants, these changes are not enough to absorb the more than 200-fold difference in specificity that FheCL1 has for these two types of substrates and the enzymes still prefer substrates with P_2_ Leu (Table 1). Previously, Stack et al. [20] found that the L67Y change in FheCL1 did not significantly modify the activity towards Tos-GPR-AMC which is consistent with our studies. However, a 13-fold increase on the activity towards this substrate was observed when a similar L67Y change was introduced into FheCL5 [38]. FheCL5 active site is more restricted at both the S_2_ and S_3_ pockets than FheCL1 due to the presence of the bulkier Leu157 and Tyr61 residues respectively. The L67Y change would impose a further restriction in the active site such that small residues at P_3_ and P_2_ would be favored. Consequently, the improved acceptance of Tos-GPR-AMC could be explained by the presence of the adjacent Gly and Pro positioned at P_3_ and P_2_ respectively, rather than by the modest rise in activity towards Pro at P_2_ as originally proposed [38]. The same rationale explains the recent observation that a FheCL5 L67F mutation increased activity towards Tos-GPR-AMC, and the inverse FheCL2 Y69L variant reduced P_2_ Pro acceptance [29].

### Functional collagenolytic assay of FhCL3 mutants

**Figure 7:**
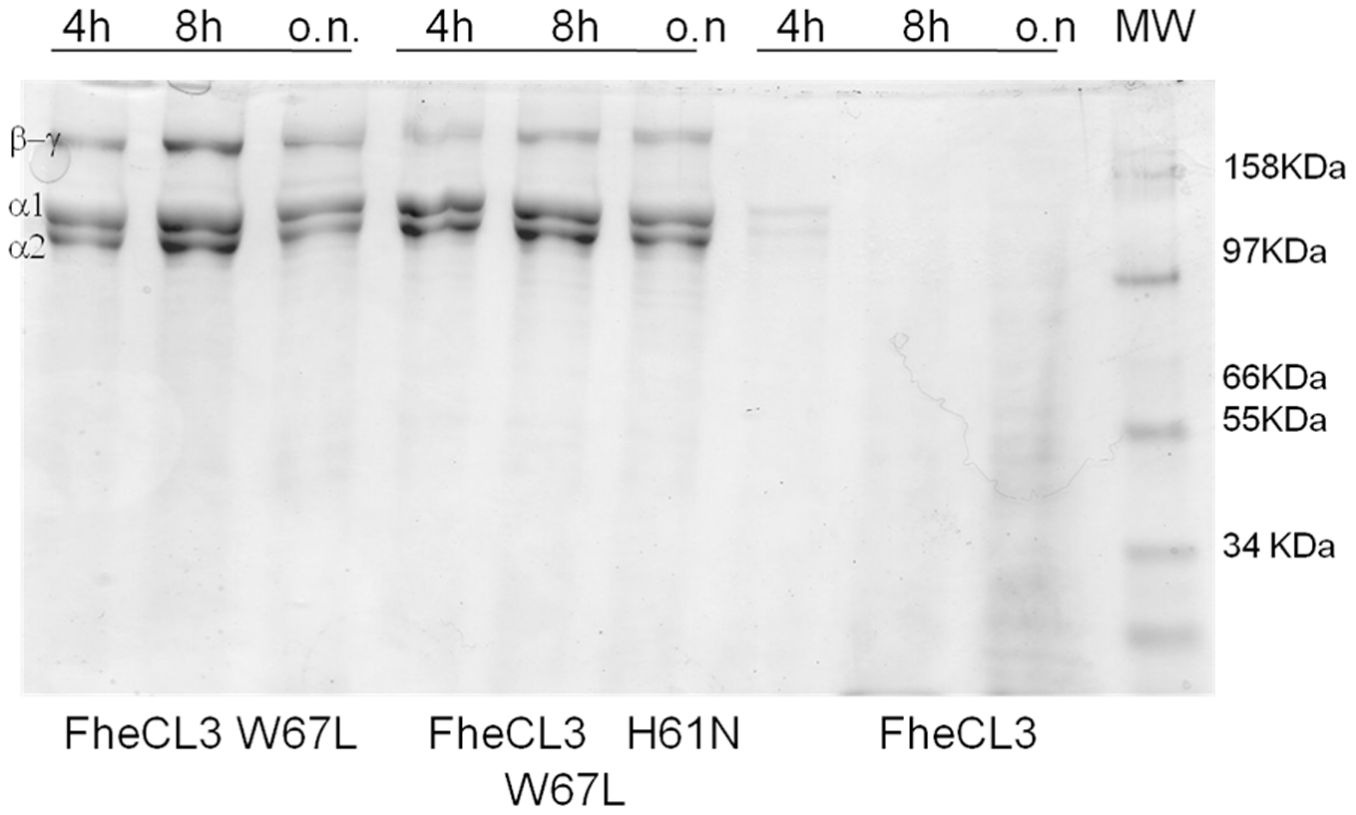
Collagen cleaving activities of recombinant *F.* *hepatica* FheCL3 and the FheCL3 enzyme variants W67L and H61N/W67L. Type I collagen was incubated with 5 µM of each enzyme for different lengths of time in PBS pH 7.3 at 28°C and separated by SDS-PAGE.

Given the unusual characteristic of FheCL3 to efficiently degrade native type I collagen, we assessed the efficacy of the parent FheCL3 and its variants to hydrolyze type I collagen *in vitro*. Unlike wild type FheCL3, both FheCL3W67L and FheCL3 H63N/W67L variants were unable to cleave collagen at neutral pH and 28°C, conditions that preserve its native structure (Figure 7). The reduced activity of FheCL3 mutants indicate that Trp67 might be crucial for the enzyme activity that might be centered in cleaving substrates enriched in small amino acids (Gly, Pro) like collagen.

Our findings agree with previous observations that the substitutions Y67L and L205A in human cathepsin K (for residues present in human cathepsin L), abolish its collagenolytic activity [37]. This human cathepsin K variant acquires the S_2_ preferences of human cathepsin L, and the reciprocal replacements to human cathepsin L conferred it with a specificity similar to cathepsin K [25]. We have also prepared a double variant of FheCL1 at the same positions, i.e. FheCL1 L67Y/L205A but this did not exhibit collagenolytic activity (data not shown). This lack of correlation with human cathepsin L and K mutants behavior is surprising, although differences at other positions within the active sites exist between the mammalian and fluke enzymes that must also be important in determining collagenolytic ability. These differences in turn might prove useful in the design of specific inhibitors or drugs for the parasite enzymes over host homologues.

### Homology modeling of FhCL3 active site

**Figure 8:**
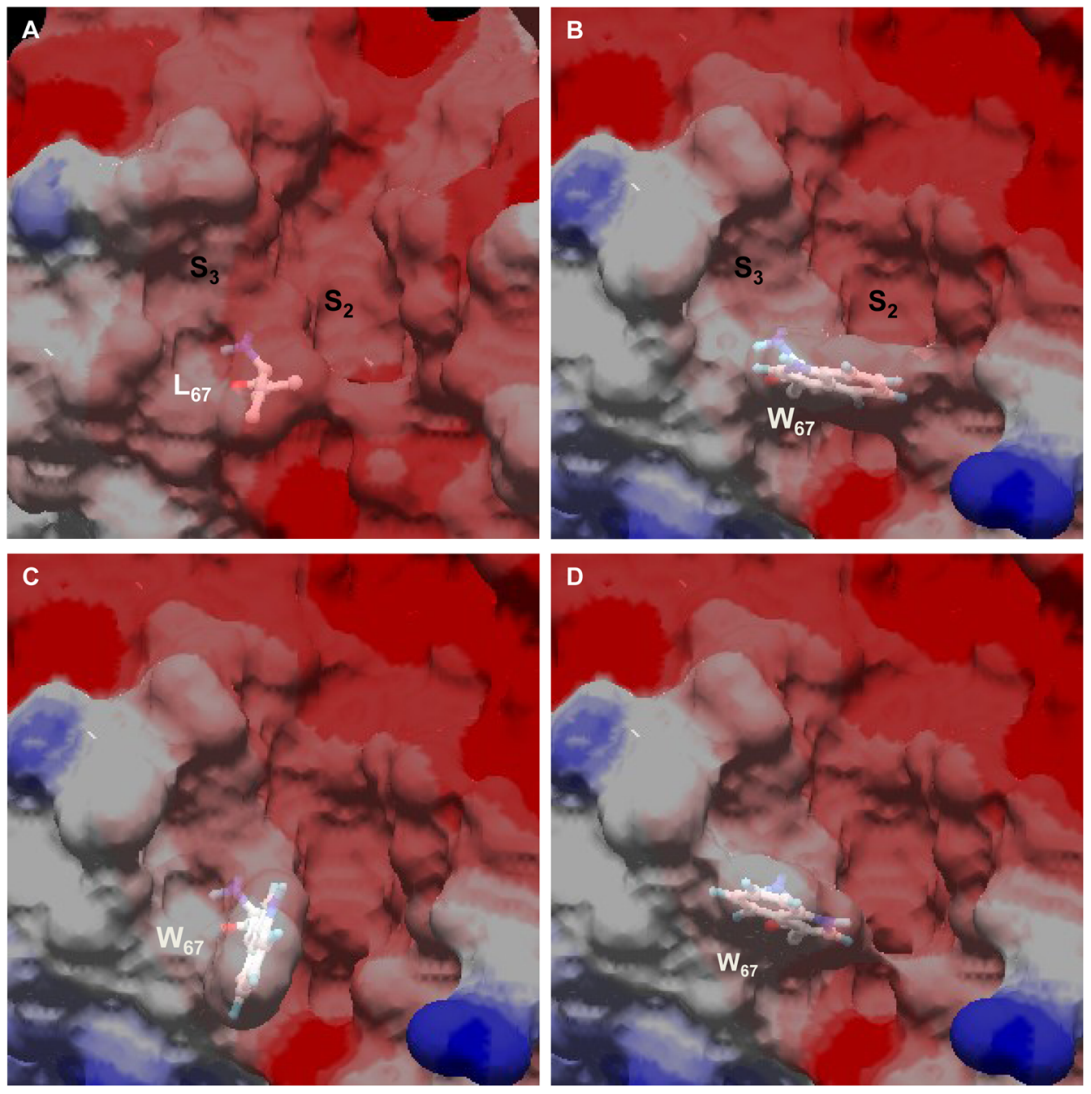
Different rotamers of Trp67 have different effects on active site pocket sizes. Comparison of FheCL1 (**A**) and FheCL3 active site pockets showing the three favoured rotamers (**B, C and D**) of Trp67 in FheCL3. The active site pockets S_2_ and S_3_ are indicated.

Our analysis of active site variants highlights the role of residue 67 which is determining by its gate-keeper position not only the conformation of the S_2_ subsite, but also of the S_3_ pocket. Using molecular modeling we analyzed the possible conformations of Trp67 in the active site of FheCL3 as compared to FheCL1 (Figure 8). The most stable rotamer protrudes and partially occludes the S_2_ subsite (Figure 8 B). An alternative conformer places the indole ring towards the S_3_ subsite reducing this site volume (Figure 8 C), while a third low energy rotamer is coaxial with the active site cleft leaving two more open but narrow active site pockets (Figure 8 D). The rotation of this residue might be fundamental to accommodating the distinct substrates of FheCL3, defining the nature of the amino acids that can be accepted in these subsites. The planar ring of Pro occupying the P_2_ subsite can be stabilized by stacking interactions with the aromatic heterocycle of Trp. Furthermore, aliphatic moieties can also be accommodated at this site due to the hydropobic nature of FheCL3 S_2_ pocket. However, at the same time than stabilizing some interactions the bulky Trp can be imposing steric hindrances in the neighbor subsite thus favoring small residues.

Based on this observation we reanalyzed the MSP-MS data looking at the amino acid pairs present at S_3_-S_2_. We noticed that FhCL3 can accommodate different residues at P_3_ if P_2_ is occupied by Pro, and that tiny Gly is slightly preferred at early times combined either with Pro or aliphatic moieties. In fact if small residues are present in P_3_, other residues can be placed in P_2_ excepting aromatic ones, which are disfavored in any combination by FheCL3 (data not shown). These combined preferences for Pro and to a lesser extent for Gly residues by FheCL3, can explain why native collagen, that is rich in these amino acids is an appropriate substrate for this enzyme.

### Conclusions

We have characterized the FheCL3 cysteine protease of the infective larval stage of *F. hepatica* that exhibits a particular collagenolytic activity and analyzed the differential contribution of active site residues involved. Our results highlight that a Trp residue strategically located at the gatekeeper position between the S_2_ and S_3_ active site pockets is vital to this activity and contributes to narrow and constrained pockets that can best accommodate small residues, particularly, Pro at P_2_ and Gly at P_3._ These peculiarities are not shared by other known cysteine proteases, suggesting that the enzyme may be a good target for the development of small molecule inhibitors for parasite control. Furthermore, our mutation analyses reveal the under-appreciated significance of interactions at P_3_ that together with those at P_2_ contribute to modulating cysteine protease specificity. Novel extended peptide libraries provide first glimpses of other interactions particularly at the prime side of the active site cleft, showing noticeable differences whose contributions to specificity and selectivity need to be assessed in future studies.

## Supporting Information

Figure S1 **Time course of peptide degradation by FheCL1 and FheCL3 analyzed by MSP-MS.** Top panel: total amount of cuts obtained by the different enzymes at 5 min, 15 min, 1 h, 4 h and 20 h incubation. Bottom panels: amino acids found at positions P_3_ and P_2_ of the cleaved peptides at different times of incubation with FheCL1 (top, blue) or FheCL3 (bottom, red) at 5, 15, and 60 min. Results are expressed as percentage per site. The amino acid frequency at each position within the tetradecapetide library ranges from 4.2% to 6.8%. Met is substituted by norleucine in the library.
(TIF)

Table S1 **Generation of active site mutants by site directed mutagenesis.** Oligonucloetide primers and templates used are indicated.
(DOCX)

## References

[1] Keiser J, Utzinger J (2009) Food-borne trematodiases. Clin Microbiol Rev 22: 466–483. 19597009 10.1128/CMR.00012-09 PMC2708390

[2] McKerrow JH, Caffrey C, Kelly B, Loke P, Sajid M (2006) Proteases in parasitic diseases. Annu Rev Pathol 1: 497–536. 18039124 10.1146/annurev.pathol.1.110304.100151

[3] Dalton J, Caffrey C, Sajid M, Stack C, Donnelly S, et al.. (2006) Proteases in trematode biology. In: Maule AG, Marks NJ, editors. Parasitic Flatworms: molecular biology, biochemistry, immunology and physiology: CAB interantional.

[4] Tort J, Brindley PJ, Knox D, Wolfe KH, Dalton JP (1999) Proteinases and associated genes of parasitic helminths. Adv Parasitol 43: 161–266. 10214692 10.1016/s0065-308x(08)60243-2

[5] Irving JA, Spithill TW, Pike RN, Whisstock JC, Smooker PM (2003) The evolution of enzyme specificity in Fasciola spp. J Mol Evol 57: 1–15. 12962301 10.1007/s00239-002-2434-x

[6] Robinson MW, Tort JF, Lowther J, Donnelly SM, Wong E, et al (2008) Proteomics and phylogenetic analysis of the cathepsin L protease family of the helminth pathogen Fasciola hepatica: expansion of a repertoire of virulence-associated factors. Mol Cell Proteomics 7: 1111–1123. 18296439 10.1074/mcp.M700560-MCP200

[7] Cancela M, Acosta D, Rinaldi G, Silva E, Duran R, et al (2008) A distinctive repertoire of cathepsins is expressed by juvenile invasive Fasciola hepatica. Biochimie 90: 1461–1475. 18573308 10.1016/j.biochi.2008.04.020

[8] Cancela M, Ruetalo N, Dell'Oca N, da Silva E, Smircich P, et al (2010) Survey of transcripts expressed by the invasive juvenile stage of the liver fluke Fasciola hepatica. BMC Genomics 11: 227. 20374642 10.1186/1471-2164-11-227 PMC2867827

[9] Morphew RM, Wright HA, Lacourse EJ, Porter J, Barrett J, et al (2011) Towards delineating functions within the fasciola secreted cathepsin l protease family by integrating in vivo based sub-proteomics and phylogenetics. PLoS Negl Trop Dis 5: e937. 21245911 10.1371/journal.pntd.0000937 PMC3014944

[10] Robinson MW, Menon R, Donnelly SM, Dalton JP, Ranganathan S (2009) An integrated transcriptomics and proteomics analysis of the secretome of the helminth pathogen Fasciola hepatica: proteins associated with invasion and infection of the mammalian host. Mol Cell Proteomics 8: 1891–1907. 19443417 10.1074/mcp.M900045-MCP200 PMC2722771

[11] Robinson MW, Dalton JP, Donnelly S (2008) Helminth pathogen cathepsin proteases: it's a family affair. Trends Biochem Sci 33: 601–608. 18848453 10.1016/j.tibs.2008.09.001

[12] Turk D, Guncar G, Podobnik M, Turk B (1998) Revised definition of substrate binding sites of papain-like cysteine proteases. Biol Chem 379: 137–147. 9524065 10.1515/bchm.1998.379.2.137

[13] Robinson MW, Corvo I, Jones PM, George AM, Padula MP, et al (2011) Collagenolytic activities of the major secreted cathepsin L peptidases involved in the virulence of the helminth pathogen, Fasciola hepatica. PLoS Negl Trop Dis 5: e1012. 21483711 10.1371/journal.pntd.0001012 PMC3071364

[14] Corvo I, Cancela M, Cappetta M, Pi-Denis N, Tort JF, et al (2009) The major cathepsin L secreted by the invasive juvenile Fasciola hepatica prefers proline in the S2 subsite and can cleave collagen. Mol Biochem Parasitol 167: 41–47. 19383516 10.1016/j.molbiopara.2009.04.005

[15] Lecaille F, Bromme D, Lalmanach G (2008) Biochemical properties and regulation of cathepsin K activity. Biochimie 90: 208–226. 17935853 10.1016/j.biochi.2007.08.011

[16] Choi KH, Laursen RA, Allen KN (1999) The 2.1 A structure of a cysteine protease with proline specificity from ginger rhizome, Zingiber officinale. Biochemistry 38: 11624–11633. 10512617 10.1021/bi990651b

[17] Kim M, Hamilton SE, Guddat LW, Overall CM (2007) Plant collagenase: unique collagenolytic activity of cysteine proteases from ginger. Biochim Biophys Acta 1770: 1627–1635. 17920199 10.1016/j.bbagen.2007.08.003

[18] Fujishima A, Imai Y, Nomura T, Fujisawa Y, Yamamoto Y, et al (1997) The crystal structure of human cathepsin L complexed with E-64. FEBS Lett 407: 47–50. 9141479 10.1016/s0014-5793(97)00216-0

[19] Pauly TA, Sulea T, Ammirati M, Sivaraman J, Danley DE, et al (2003) Specificity determinants of human cathepsin s revealed by crystal structures of complexes. Biochemistry 42: 3203–3213. 12641451 10.1021/bi027308i

[20] Stack CM, Caffrey CR, Donnelly SM, Seshaadri A, Lowther J, et al (2008) Structural and functional relationships in the virulence-associated cathepsin L proteases of the parasitic liver fluke, Fasciola hepatica. J Biol Chem 283: 9896–9908. 18160404 10.1074/jbc.M708521200 PMC3979170

[21] Zhao B, Janson CA, Amegadzie BY, D'Alessio K, Griffin C, et al (1997) Crystal structure of human osteoclast cathepsin K complex with E-64. Nat Struct Biol 4: 109–111. 9033588 10.1038/nsb0297-109

[22] Kim MJ, Yamamoto D, Matsumoto K, Inoue M, Ishida T, et al (1992) Crystal structure of papain-E64-c complex. Binding diversity of E64-c to papain S2 and S3 subsites. Biochem J 287 (Pt 3) 797–803. 1445241 10.1042/bj2870797 PMC1133078

[23] O'Hara BP, Hemmings AM, Buttle DJ, Pearl LH (1995) Crystal structure of glycyl endopeptidase from Carica papaya: a cysteine endopeptidase of unusual substrate specificity. Biochemistry 34: 13190–13195. 7548082 10.1021/bi00040a034

[24] Schroder E, Phillips C, Garman E, Harlos K, Crawford C (1993) X-ray crystallographic structure of a papain-leupeptin complex. FEBS Lett 315: 38–42. 8416808 10.1016/0014-5793(93)81128-m

[25] Lecaille F, Chowdhury S, Purisima E, Bromme D, Lalmanach G (2007) The S2 subsites of cathepsins K and L and their contribution to collagen degradation. Protein Sci 16: 662–670. 17384231 10.1110/ps.062666607 PMC2203344

[26] O'Donoghue AJ, Eroy-Reveles AA, Knudsen GM, Ingram J, Zhou M, et al (2012) Global identification of peptidase specificity by multiplex substrate profiling. Nat Methods 2012: 1095–100. 10.1038/nmeth.2182 PMC3707110 23023596

[27] Choe Y, Leonetti F, Greenbaum DC, Lecaille F, Bogyo M, et al (2006) Substrate profiling of cysteine proteases using a combinatorial peptide library identifies functionally unique specificities. J Biol Chem 281: 12824–12832. 16520377 10.1074/jbc.M513331200

[28] Harris JL, Backes BJ, Leonetti F, Mahrus S, Ellman JA, et al (2000) Rapid and general profiling of protease specificity by using combinatorial fluorogenic substrate libraries. Proc Natl Acad Sci U S A 97: 7754–7759. 10869434 10.1073/pnas.140132697 PMC16617

[29] Norbury LJ, Hung A, Beckham S, Pike RN, Spithill TW, et al (2012) Analysis of Fasciola cathepsin L5 by S2 subsite substitutions and determination of the P1–P4 specificity reveals an unusual preference. Biochimie 94: 1119–1127. 22285967 10.1016/j.biochi.2012.01.011

[30] Faber KN, Haima P, Harder W, Veenhuis M, Ab G (1994) Highly-efficient electrotransformation of the yeast Hansenula polymorpha. Curr Genet 25: 305–310. 8082173 10.1007/BF00351482

[31] Smith PK, Krohn RI, Hermanson GT, Mallia AK, Gartner FH, et al (1985) Measurement of protein using bicinchoninic acid. Anal Biochem 150: 76–85. 3843705 10.1016/0003-2697(85)90442-7

[32] Arnold K, Bordoli L, Kopp J, Schwede T (2006) The SWISS-MODEL workspace: a web-based environment for protein structure homology modelling. Bioinformatics 22: 195–201. 16301204 10.1093/bioinformatics/bti770

[33] Guex N, Peitsch MC (1997) SWISS-MODEL and the Swiss-PdbViewer: an environment for comparative protein modeling. Electrophoresis 18: 2714–2723. 9504803 10.1002/elps.1150181505

[34] Portaro FC, Santos AB, Cezari MH, Juliano MA, Juliano L, et al (2000) Probing the specificity of cysteine proteinases at subsites remote from the active site: analysis of P4, P3, P2' and P3' variations in extended substrates. Biochem J 347 Pt 1: 123–129. 10727410 PMC1220939

[35] Menard R, Carmona E, Plouffe C, Bromme D, Konishi Y, et al (1993) The specificity of the S1' subsite of cysteine proteases. FEBS Lett 328: 107–110. 8344413 10.1016/0014-5793(93)80975-z

[36] Alves MF, Puzer L, Cotrin SS, Juliano MA, Juliano L, et al (2003) S3 to S3' subsite specificity of recombinant human cathepsin K and development of selective internally quenched fluorescent substrates. Biochem J 373: 981–986. 12733990 10.1042/BJ20030438 PMC1223542

[37] Lecaille F, Choe Y, Brandt W, Li Z, Craik CS, et al (2002) Selective inhibition of the collagenolytic activity of human cathepsin K by altering its S2 subsite specificity. Biochemistry 41: 8447–8454. 12081494 10.1021/bi025638x

[38] Smooker PM, Whisstock JC, Irving JA, Siyaguna S, Spithill TW, et al (2000) A single amino acid substitution affects substrate specificity in cysteine proteinases from Fasciola hepatica. Protein Sci 9: 2567–2572. 11206078 10.1110/ps.9.12.2567 PMC2144524

