# Supplementary figures and images for: Dissecting the Active Site of the Collagenolytic Cathepsin L3 Protease of the Invasive Stage of Fasciola hepatica
Source: PLoS Negl Trop Dis. 2013 Jul 11;7(7):e2269. doi: 10.1371/journal.pntd.0002269 (PMC3708847; doi:10.1371/journal.pntd.0002269)

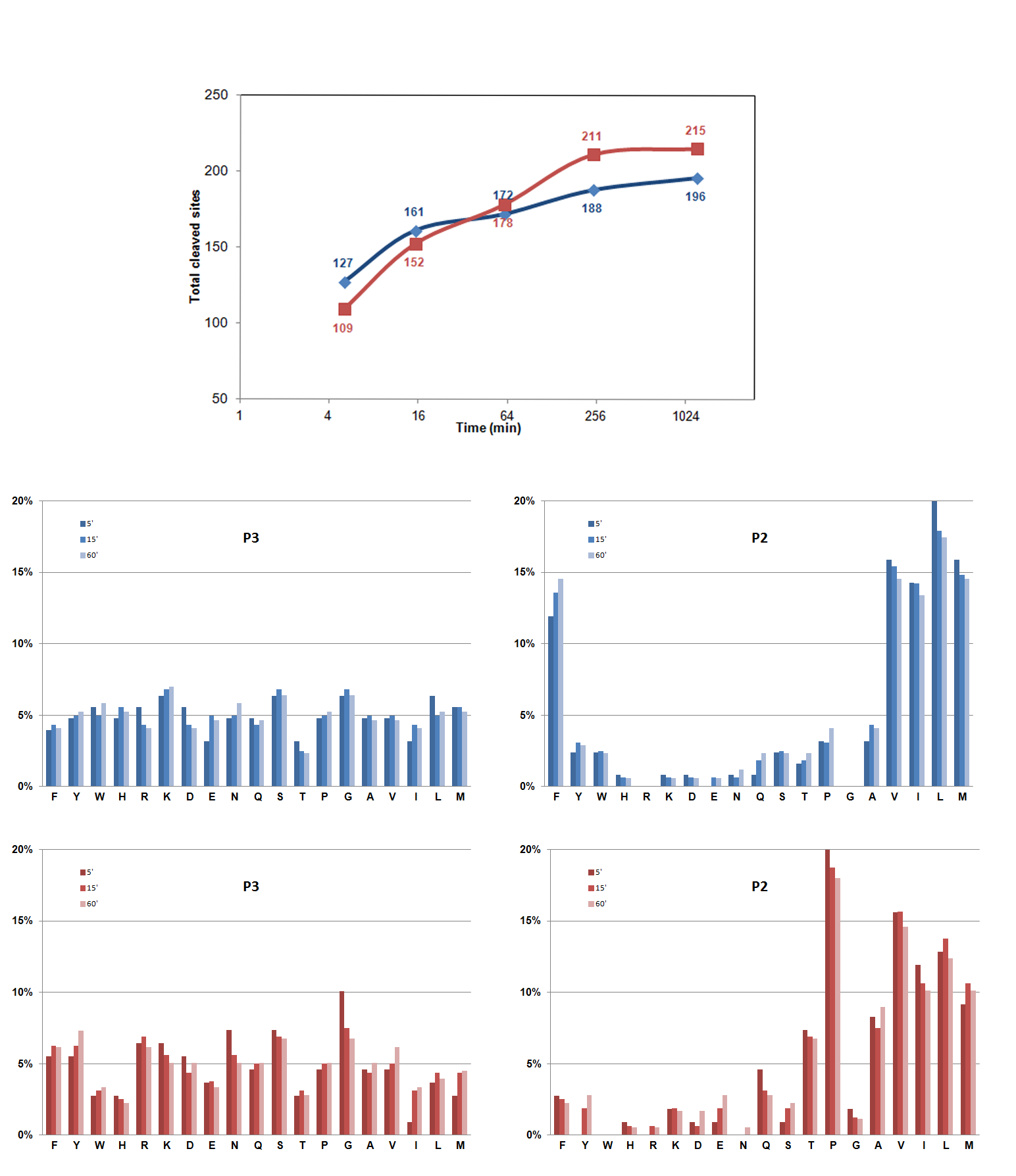

Supplement: Figure S1 — Time course of peptide degradation by FheCL1 and FheCL3 analyzed by MSP-MS. Top panel: total amount of cuts obtained by the different enzymes at 5 min, 15 min, 1 h, 4 h and 20 h incubation. Bottom panels: amino acids found at positions P3 and P2 of the cleaved peptides at different times of incubation with FheCL1 (top, blue) or FheCL3 (bottom, red) at 5, 15, and 60 min. Results are expressed as percentage per site. The amino acid frequency at each position within the tetradecapetide library ranges from 4.2% to 6.8%. Met is substituted by norleucine in the library. (TIF) [file pntd.0002269.s001.tif]
