# Supplementary material for: Dissecting the Active Site of the Collagenolytic Cathepsin L3 Protease of the Invasive Stage of Fasciola hepatica
Source: PLoS Negl Trop Dis. 2013 Jul 11;7(7):e2269. doi: 10.1371/journal.pntd.0002269 (PMC3708847; doi:10.1371/journal.pntd.0002269)
Supplement: Table S1 — Generation of active site mutants by site directed mutagenesis. Oligonucloetide primers and templates used are indicated. (DOCX) [file pntd.0002269.s002.docx]

**TABLE S1**

**Generation of active site mutants by site directed mutagenesis.**

| **Enzyme variant** | **Plasmid Template** | **Oligonucleotide** | **Sequence*** |
| --- | --- | --- | --- |
| FheCL3 H61N | FheCL3 wild type | FheCL3 H61N Fw | CAGAAGATTTGGCAAC***AAC***GGATGTGGAGGTGG |
|  |  | FheCL3 H61N Rv | CCACCTCCACATCC***GTT***GTTGCCAAATCTTCTG |
| FheCL3 W67L | FheCL3 wild type | FheCL3 W67L Fw | CGGATGTGGAGGTGG***TTT***GATGGAGAACGC |
|  |  | FheCL3 W67L Rv | GCGTTCTCCAT***CAA***ACCACCTCCACATCCG |
| FheCL3 H61N W67L | FheCL3 H61N | FheCL3 W67L Fw & Rv |  |
| FheCL1 N61H | FheCL1 wild type | FheCL1 N61H Fw | CGACCTTGGGGAAAT***CAT***GGTTGCGGTGG |
|  |  | FheCL1 N61H Rv | CCACCGCAACC***ATG***ATTTCCCCAAGGTCG |
| FheCL1 L67W | FheCL1 wild type | FheCL1 L67W Fw | GTTGCGGTGGTGGA***TGG***ATGGAAAATGCTTACC |
|  |  | FheCL1 L67W Rv | GGTAAGCATTTTCCAT***CCA***TCCACCACCGCAAC |
| FheCL1 N61H L67W | FhCL1 N61H | FheCL1 L67W Fw & Rv |  |

* Residues introducing the desired mutation are indicated in italics bold
